# Supplementary material for: Real-Time Integrative Mapping of the Phenology and Climatic Suitability for the Spotted Lanternfly, Lycorma delicatula
Source: Insects. 2025 Jul 31;16(8):790. doi: 10.3390/insects16080790 (PMC12386670; doi:10.3390/insects16080790)
Supplement: Supplementary file 1 [file insects-16-00790-s001.zip › insects-3707996-supplementary.pdf]

Supplementary Material

Real-Time Integrative Mapping of the Phenology and Climatic Suitability for the Spotted Lanternfly, *Lycorma delicatula*

Brittany S. Barker [1,2,*], Jules Beyer [1], and Leonard Coop [1,2]

[1] Oregon Integrated Pest Management Center, Oregon State University, 2215 Cordley Hall, Corvallis, OR 97331, USA; beyerju@oregonstate.edu (J.B.); coopl@oregonstate.edu (L.C.)

[2] Department of Horticulture, Oregon State University, 4017 Agriculture and Life Sciences Building, Corvallis, OR 97331, USA

* Correspondence: brittany.barker@oregonstate.edu

Figure S1. Histogram showing the distribution of dates (day of year, DOY) for egg observations in the *iNaturalist* dataset for spotted lanternfly. Observations were assigned to the overwintered (OW) generation (blue shading) vs. first generation (G1, salmon shading) for the model validation analysis.

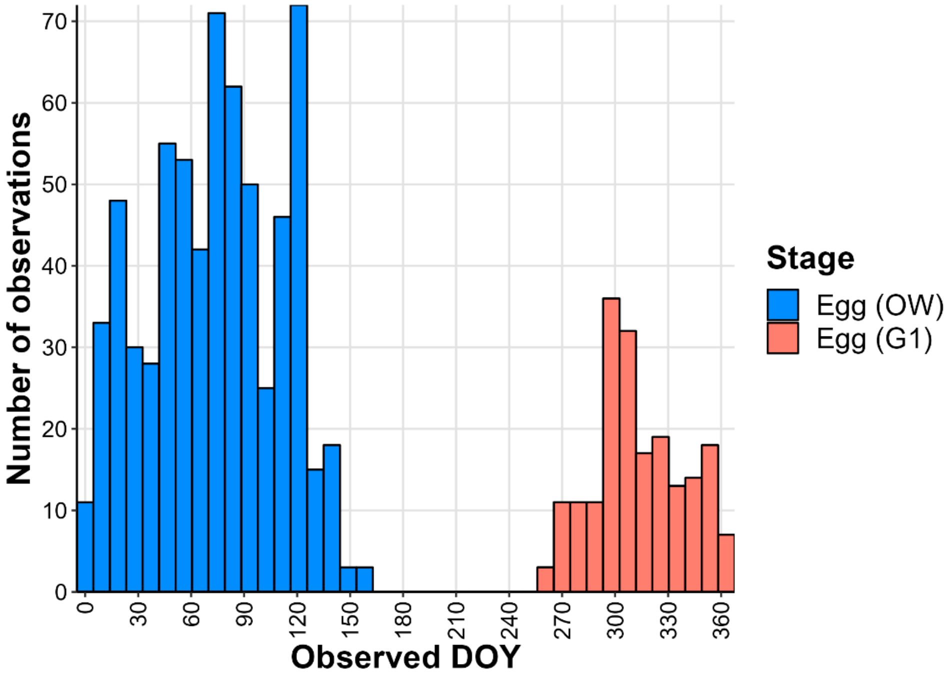

Figure S2. Maps depicting consecutive cold days and model-predicted cold stress accumulation for spotted lanternfly for China. Maps of (A) consecutive days of minimum temperatures (T_{min}) below –16 °C based on 20-year climate averages centered on 2008 (1999–2018), (B) annual cold stress accumulation based on 20-year climate averages, and (C) annual cold stress accumulation based on climate data for an extreme year in terms of cold stress accumulation (2001) were used to calibrate moderate and severe cold stress limits in the DDRP model. Presence records are depicted in map A.

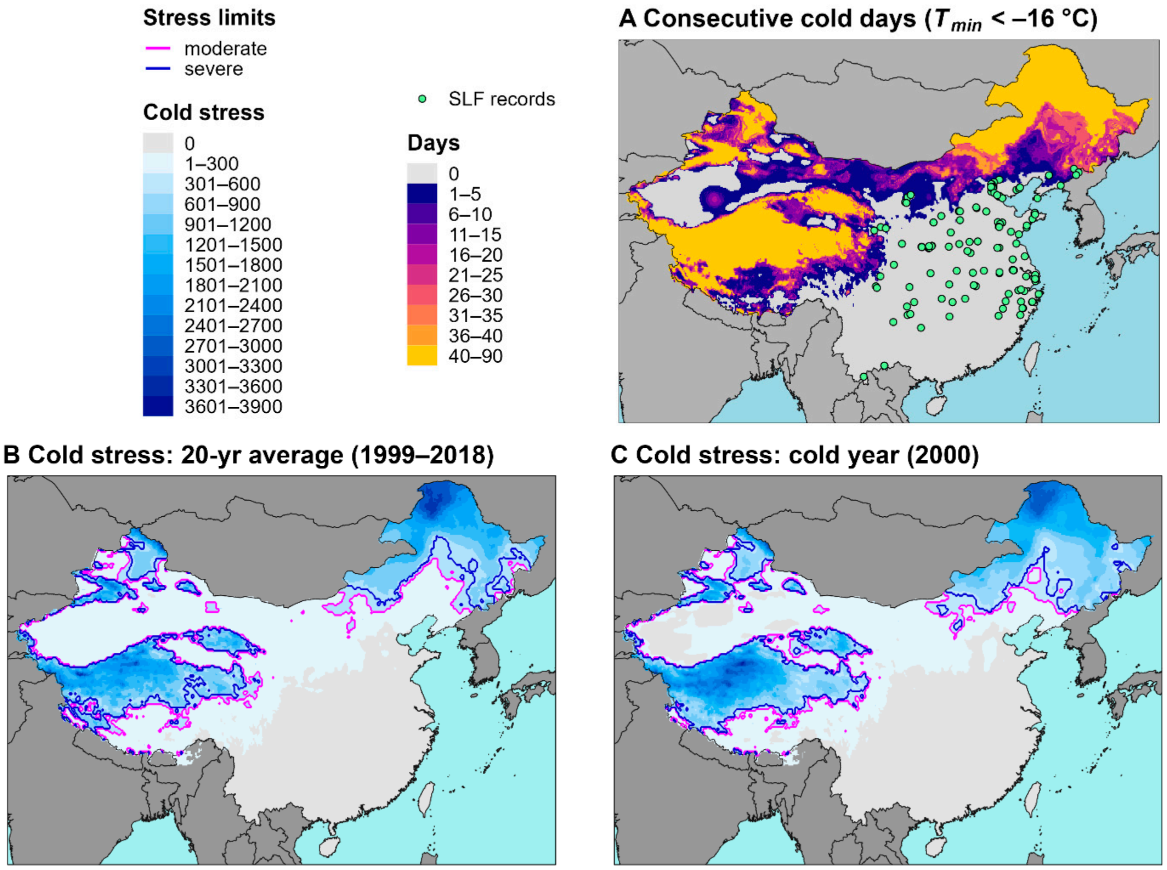

Figure S3. Maps depicting consecutive hot days and model-predicted heat stress accumulation for spotted lanternfly for China. Maps of (A) consecutive days of maximum temperatures (T_{max}) above 37 °C based on 20-year climate averages centered on 2008 (1999−2018), (B) annual heat stress accumulation based on 20-year climate averages, and (C) annual heat stress accumulation based on climate data for an extreme year in terms of heat stress accumulation (2008) were used to calibrate moderate and severe heat stress limits in the DDRP model. Presence records are depicted in map A.

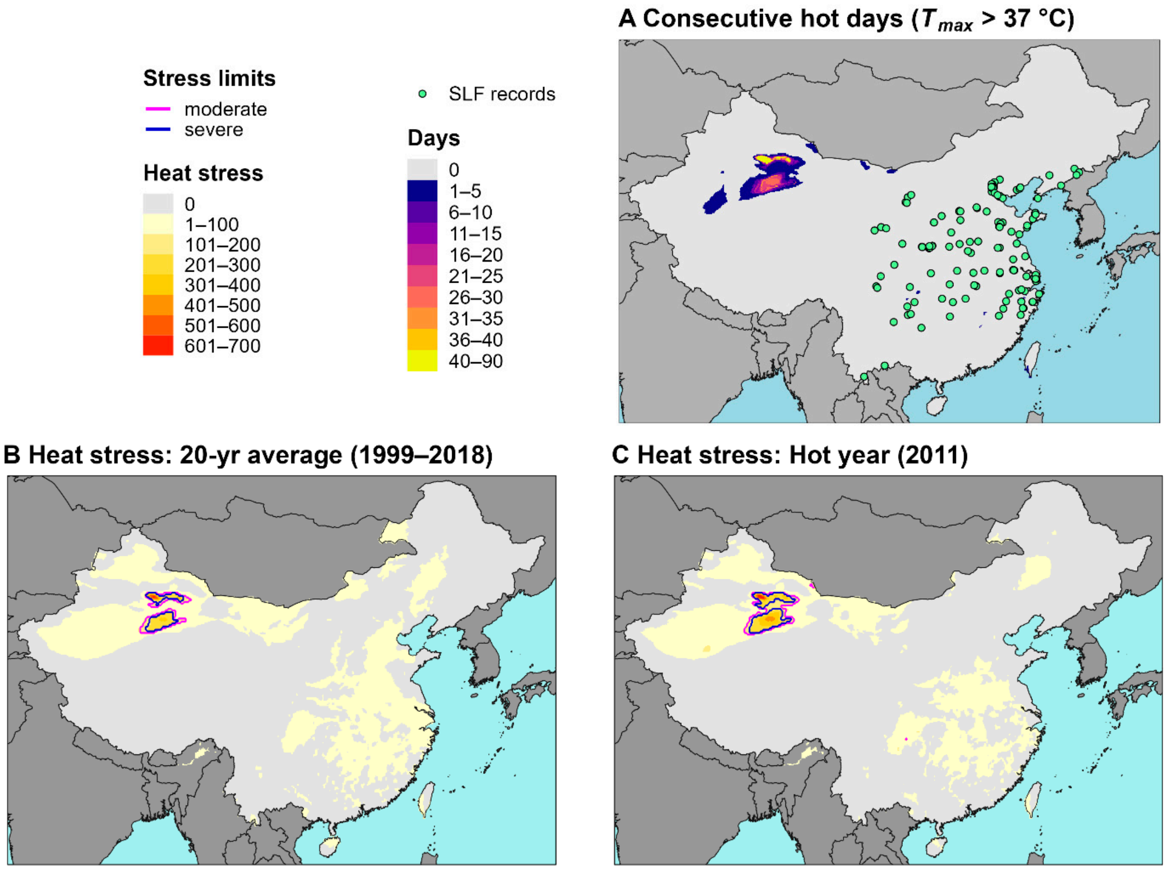

Table S1. Error rates of DDRP models for spotted lanternfly that applied different cohort parameter values. Parameters include the minimum (*xdist1*), mean (*distro_mean*), and maximum (*xdist2*) degree-days (DDC) to complete egg development. The mean absolute error (MAE), bias (± standard deviation, SD), and number of over-predicted dates (in days) are indicated for each phenological event (first egg hatch and peak egg hatch). Bias was calculated as the average amount by which predicted days of the year (DOYs) are greater than observed DOYs. Negative bias values may indicate model underprediction (too early) whereas positive values may indicate overprediction (too late). Parameter set 7 (bold font) was used in the final model.

Set	Event	xdist1	mean	xdist2	MAE	Bias ± SD	No. over-predicted
1	first egg hatch	180	240	400	7.4	1.9 ± 9.1	4
1	peak egg hatch	180	240	400	7.0	2.4 ± 8.4	4
2	first egg hatch	170	240	400	6.4	–0.6 ± 8.0	4
2	peak egg hatch	170	240	400	6.4	1.3 ± 8.1	4
3	first egg hatch	170	230	380	6.4	–0.6 ± 8.0	4
3	peak egg hatch	170	230	380	6.3	0.3 ± 8.1	3
4	first egg hatch	160	220	360	6.0	–2.8 ± 7.6	3
4	peak egg hatch	160	220	360	6.6	–1.1 ± 8.1	3
5	first egg hatch	150	215	360	7.2	–4.8 ± 8.1	3
5	peak egg hatch	150	215	360	6.6	–2.0 ± 7.9	3
6	first egg hatch	140	215	360	7.8	–6.8 ± 7.4	1
6	peak egg hatch	140	215	360	6.7	–3.0 ± 7.9	3
**7**	**first egg hatch**	**135**	**190**	**360**	**8.0**	**–8.0 ± 7.1**	**0**
**7**	**peak egg hatch**	**135**	**190**	**360**	**6.7**	**–3.9 ± 7.8**	**2**

Table S2. Comparison of predicted vs. observed dates (day of year, DOY) of first and peak egg hatch for overwintered eggs of spotted lanternfly based on the cohort parameter values used in the final model. The geographic origin (state, site, latitude, and longitude), phenological event, date, day of year (DOY_{obs}), and source (Ref) of each observation is provided. Differences in days between model-predicted DOY (DOY_{mod}) and DOY_{obs} (Diff = predicted DOY– observed DOY) were calculated for each observation.

State	Site	Lat	Lon	Event	Date	Obs. DOY	Pred. DOY	Diff	Ref
PA	Boyertown	-75.637	40.334	first egg hatch	5/23/2016	144	131	–13	1
PA	Boyertown	-75.637	40.334	peak egg hatch	6/5/2016	157	145	–12	1
PA	Oley	-75.711	40.405	first egg hatch	5/1/2017	121	120	–1	2
PA	Oley	-75.711	40.405	first egg hatch	5/1/2017	121	120	–1	3
PA	Oley	-75.711	40.405	peak egg hatch	5/15/2017	135	139	4	2
PA	Oley	-75.711	40.405	peak egg hatch	5/23/2017	143	139	–4	3
VA	Winchester	-78.155	39.206	first egg hatch	4/30/2019	124	116	–8	3
PA	Winchester	-78.155	39.206	first egg hatch	5/1/2019	121	116	–5	4
VA	Winchester	-78.155	39.206	peak egg hatch	5/7/2019	134	128	–6	3
PA	Winchester	-78.155	39.206	peak egg hatch	5/8/2019	128	128	0	4
VA	Winchester	-78.155	39.206	first egg hatch	5/10/2019	130	116	–14	5
PA	Kutztown	-75.777	40.517	first egg hatch	5/23/2019	143	123	–20	6
PA	Kutztown	-75.777	40.517	peak egg hatch	6/1/2019	152	137	–15	6
PA	Winchester	-78.155	39.206	first egg hatch	5/7/2020	128	126	–2	4
PA	Winchester	-78.155	39.206	peak egg hatch	5/16/2020	137	143	6	4

References

1. Murman, K., G. P. Setliff, C. V. Pugh, M. J. Toolan, et al. Distribution, survival, and development of spotted lanternfly on host plants found in North America. *Environ Entomol* (2020) 49:1270–1281. doi: 10.1093/ee/nvaa126
2. Liu, H. Oviposition substrate selection, egg mass characteristics, host preference, and life history of the spotted lanternfly (Hemiptera: Fulgoridae) in North America. *Environ Entomol* (2019) 48:1452–1468. 10.1093/ee/nvz123
3. Smyers, E. C., J. M. Urban, A. C. Dechaine, D. G. Pfeiffer, S. R. et al. Spatio-temporal model for predicting spring hatch of the spotted lanternfly (Hemiptera: Fulgoridae). *Environ Entomol* (2021) 50:126–137. doi: 10.1093/ee/nvaa129
4. Dechaine, A. C., M. Sutphin, T. C. Leskey, S. M. Salom, et al. 2021. Phenology of *Lycorma delicatula* (Hemiptera: Fulgoridae) in Virginia, USA. *Environ Entomol* (2021) 50:1267–1275. doi: 10.1093/ee/nvab107
5. Nixon, L. J., H. Leach, C. Barnes, J. Urban, et al. Development of behaviorally based monitoring and biosurveillance tools for the invasive spotted lanternfly (Hemiptera: Fulgoridae). *Environ Entomol* (2020) 49:1117–1126. doi: 10.1093/ee/nvaa084
6. Leach, H., and A. Leach. Seasonal phenology and activity of spotted lanternfly (*Lycorma delicatula*) in eastern US vineyards. *J Pest Sci* (2020) 93:1215–1224. doi: 10.1007/s10340-020-01233-7

Table S3. Field observations of phenological events derived from the literature used to validate the DDRP model for spotted lanternfly (SLF). The geographic origin (state, site, latitude, and longitude), life stage, event type, date, day of year (DOY_{obs}), and source (Ref) of each observation is provided. Differences in days between model-predicted DOY (DOY_{pred}) and DOY_{obs} ($Diff = DOY_{pred} - DOY_{obs}$) were calculated for first dates of egg hatch, nymphs halfway developed, appearance of adults, and oviposition (coded in the model as e0, l0, p0, and a0, respectively). Coordinate data for Warren County, NJ, were estimated as the average latitude (Lat) and longitude (Lon) across five plots. Host species used by SLF varied across monitoring sites.

State	Site	Lat	Lon	Stage	Event	PEM	Year	Date	DOY_{obs}	DOY_{pred}	Diff	Ref
PA	Mohnton	40.286	-75.984	early nymph	egg hatch	e0	2020	07/07/2020	189	140	-49	1
PA	Mohnton	40.286	-75.984	early nymph	egg hatch	e0	2021	06/09/2021	160	124	-36	1
PA	Mohnton	40.286	-75.984	early nymph	egg hatch	e0	2022	05/18/2022	138	132	-6	1
PA	Wyomissing	40.329	-75.965	early nymph	egg hatch	e0	2019	06/06/2019	157	123	-34	2
PA	Wyomissing	40.329	-75.965	early nymph	egg hatch	e0	2020	06/01/2020	153	139	-14	2
PA	Mohnton	40.286	-75.984	late nymph	nymphs halfway devel.	l0	2020	07/10/2020	192	181	-11	1
PA	Mohnton	40.286	-75.984	late nymph	nymphs halfway devel.	l0	2021	06/23/2021	174	175	1	1
PA	Mohnton	40.286	-75.984	late nymph	nymphs halfway devel.	l0	2022	06/15/2022	166	175	9	1
PA	Wyomissing	40.329	-75.965	late nymph	nymphs halfway devel.	l0	2019	06/25/2019	176	173	-3	2
PA	Wyomissing	40.329	-75.965	late nymph	nymphs halfway devel.	l0	2020	06/22/2020	174	180	6	2
PA	Boyertown	40.334	-75.637	adult	appearance of adults	p0	2017	08/03/2017	215	209	-6	3
PA	Mohnton	40.286	-75.984	adult	appearance of adults	p0	2020	07/30/2020	212	211	-1	1
PA	Mohnton	40.286	-75.984	adult	appearance of adults	p0	2021	08/04/2021	216	207	-9	1
PA	Mohnton	40.286	-75.984	adult	appearance of adults	p0	2022	07/26/2022	207	207	0	1
PA	Norristown	40.128	-75.34	adult	appearance of adults	p0	2020	07/23/2020	205	208	3	4
PA	Norristown	40.128	-75.34	adult	appearance of adults	p0	2021	08/02/2021	214	203	-11	4
PA	Norristown	40.128	-75.34	adult	appearance of adults	p0	2022	07/28/2022	209	204	-5	4
PA	Norristown	40.128	-75.34	adult	appearance of adults	p0	2023	07/25/2023	206	208	2	4
PA	Penn Township	40.415	-76.096	adult	appearance of adults	p0	2019	08/01/2019	213	206	-7	4
NJ	Warren County	40.94	-74.97	adult	appearance of adults	p0	2020	08/10/2020	223	219	-4	5
NJ	Warren County	40.94	-74.97	adult	appearance of adults	p0	2021	08/15/2021	227	221	-6	5
PA	Wyomissing	40.329	-75.965	adult	appearance of adults	p0	2019	07/23/2019	204	204	0	2
PA	Wyomissing	40.329	-75.965	adult	appearance of adults	p0	2020	07/27/2020	209	210	1	2
PA	Boyertown	40.334	-75.637	egg	oviposition	a0	2017	10/04/2017	277	269	-8	3

State	Site	Lat	Lon	Stage	Event	PEM	Year	Date	DOY_{obs}	DOY_{pred}	Diff	Ref
NJ	Warren County	40.94	-74.97	egg	oviposition	a0	2020	09/16/2020	260	297	37	5
NJ	Warren County	40.94	-74.97	egg	oviposition	a0	2021	09/20/2021	263	282	19	5
PA	Wyomissing	40.329	-75.965	egg	oviposition	a0	2019	09/25/2019	268	251	-17	2
PA	Wyomissing	40.329	-75.965	egg	oviposition	a0	2020	09/28/2020	272	255	-17	2

References

1. Deecher, E. M. Monitoring and cold tolerance of the invasive spotted lanternfly, *Lycorma delicatula* (Hemiptera: Fulgoridae). University Park, PA: Pennsylvania State University (2023). Master's Thesis.

2. Calvin, D. D., J. Rost, J. Keller, S. Crawford, et al. Seasonal activity of spotted lanternfly (Hemiptera: Fulgoridae), in Southeast Pennsylvania. *Environ Entomol* (2023) 52:1108-1125. doi: 10.1093/ee/nvad093

3. Baker, T. C., E. C. Smyers, J M. Urban, Z. Meng, et al. Progression of seasonal activities of adults of the spotted lanternfly, *Lycorma delicatula*, during the 2017 season of mass flight dispersal behavior in eastern Pennsylvania. *J Asia-Pac Entomol* (2019) 22:705−713. doi: 10.1016/j.aspen.2019.05.006

4. Natures Notebook Database. Data download using the *rnpn* R package v. 1.2.7 (2025) [Accessed Jan 4, 2025].

5. Cooperband, J. F., and K. Murman. Responses of adult spotted lanternflies to artificial aggregations composed of all males or females. *Front Insect Sci* (2022) 2:981832. doi: 10.3389/finsc.2022.981832

Table S4. Results of the validation analysis that used *iNaturalist* observations for spotted lanternfly for (A) the United States and (B) China. Life stages predicted by DDRP are shown in relation to observed stages. A stage that was predicted later than it was observed (Stage = Later) may indicate model overprediction (except for overwintered eggs because the model starts with this stage). The number of observations corresponding to each predicted stage (N), total sample size for observed stage (Total N), and percentage of observations predicted to be in each stage [Perc = (N / Total N) × 100] (Perc) are indicated. OW = overwintered, G1 = first generation.

(B) United States

Observed	Predicted	N	Total N	Perc	Stage
Egg (OW)	Egg (OW)	332	334	99.4	Same
Egg (OW)	Early nymph (last egg hatch)	2	334	0.6	−
Early nymph	Egg (OW)	46	2561	1.8	Later
Early nymph	Early nymph (first egg hatch)	1694	2561	66.1	Same
Early nymph	Late nymph (first nymphs halfway devel.)	801	2561	31.3	Earlier
Early nymph	Adult (first appearance of adults)	13	2561	0.5	Earlier
Early nymph	Post-oviposition	7	2561	0.3	Earlier
Late nymph	Egg (OW)	0	2285	0	Later
Late nymph	Early nymph (first egg hatch)	13	2065	0.6	Later
Late nymph	Late nymph (first nymphs halfway devel.)	1617	2065	78.3	Same
Late nymph	Adult (first appearance of adults)	423	2065	20.5	Earlier
Late nymph	Post-oviposition	12	2065	0.6	Earlier
Adult	Egg (OW)	43	7806	0.6	Later
Adult	Early nymph (first egg hatch)	12	7806	0.2	Later
Adult	Late nymph (first nymphs halfway devel.)	187	7806	2.4	Later
Adult	Adult (first appearance of adults)	3714	7806	47.6	Same
Adult	Post-oviposition	3850	7806	49.3	Earlier
Egg (G1)	Egg (OW)	0	161	0	Later
Egg (G1)	Early nymph (first egg hatch)	0	161	0	Later
Egg (G1)	Late nymph (first nymphs halfway devel.)	0	161	0	Later
Egg (G1)	Adult (first appearance of adults)	5	161	3.1	Later
Egg (G1)	Post-oviposition	156	161	96.9	Same

(B) China

Observed	Predicted	N	Total N	Perc	Stage
Early nymph	Early nymph (first egg hatch)	1	4	25	Same
Early nymph	Late nymph (first nymphs halfway devel.)	3	4	75	Earlier
Late nymph	Early nymph (first egg hatch)	1	10	10	Later
Late nymph	Late nymph (first nymphs halfway devel.)	4	10	40	Same
Late nymph	Adult (first appearance of adults)	5	10	50	Earlier
Adult	Adult (first appearance of adults)	14	41	34.1	Same
Adult	Post-oviposition	27	41	65.9	Earlier
